# Investigation into the causes of indwelling urethral catheter implementation and its effects on clinical outcomes and health care resources among dementia patients with pneumonia: A retrospective cohort study: Erratum

**DOI:** 10.1097/01.md.0000503455.40174.9b

**Published:** 2016-10-07

In the article “Investigation into the causes of indwelling urethral catheter implementation and its effects on clinical outcomes and health care resources among dementia patients with pneumonia: A retrospective cohort study”,^[1]^ which appeared in Volume 95, Issue 35 of *Medicine*, the term “univariate” appeared incorrectly as “bivariate” throughout the original article. The article has since been corrected online.
